# Supplementary material for: Rapid development of neutralizing and diagnostic SARS-COV-2 mouse monoclonal antibodies
Source: Sci Rep. 2021 May 6;11:9682. doi: 10.1038/s41598-021-88809-0 (PMC8102525; doi:10.1038/s41598-021-88809-0)
Supplement: Supplementary file 1 — Supplementary Information. [file 41598_2021_88809_MOESM1_ESM.pdf]

Supplementary Information

Rapid Development of Neutralizing and Diagnostic SARS-COV-2 Mouse Monoclonal Antibodies

Asheley P. Chapman,[1] Xiaoling Tang,[2‡] Joo R. Lee,[2‡] Asiya Chida,[2‡] Kristina Mercer,[3] Rebekah E. Wharton,[3] Markus Kainulainen,[4] Jennifer L. Harcourt,[5] Roosecelis B. Martines,[6] Michelle Schroeder,[1] Liangjun Zhao,[1] Anton Bryksin,[7] Bin Zhou,[8] Eric Bergeron,[4] Brigid C. Bollweg,[6] Azaibi Tamin,[5] Natalie Thornburg,[5] David E. Wentworth,[8] David Petway,[2] Dennis Bagarozzi, Jr,[2] M.G. Finn,[1,9]* Jason M. Goldstein[2]*

[1] School of Chemistry and Biochemistry, Georgia Institute of Technology, 901 Atlantic Dr., Atlanta, GA, USA, 30306

[2] Immunodiagnostic Development Team/ Reagent Diagnostic Services Branch (RDSB)/DSR/NCEZID/CDC, 1600 Clifton Rd NE. Atlanta, GA, USA, 30333

[3] Division of Laboratory Sciences DLS/NCEH/CDC, 4770 Buford Hwy, Atlanta, GA 30341, USA

[4] Viral Special Pathogens Branch VSPB/DHCPP/NCEZID/CDC, 1600 Clifton Rd NE. Atlanta, GA, USA, 30333

[5] Respiratory Disease Branch (RDB)/DVD/NCIRD/CDC, 1600 Clifton Rd NE. Atlanta, GA, USA, 30333

[6] Infectious Disease Pathology Branch (IDPB)/DHCPP/NCEZID/CDC, 1600 Clifton Rd NE. Atlanta, GA, USA, 30333

[7] Parker H. Petit Institute for Bioengineering and Bioscience, Georgia Institute of Technology, Atlanta, GA, USA, 30306

[8] Vaccine Preparedness Team/ Virology Surveillance and Diagnosis Branch (VSPB)/ID/NCIRD/CDC, 1600 Clifton Rd NE. Atlanta, GA, USA, 30333

[9] School of Biological Sciences, Georgia Institute of Technology, 901 Atlantic Dr., Atlanta, GA, USA, 30306

*Corresponding authors: mgfinn@gatech.edu, fex0@cdc.gov

‡Authors provided equivalent contributions

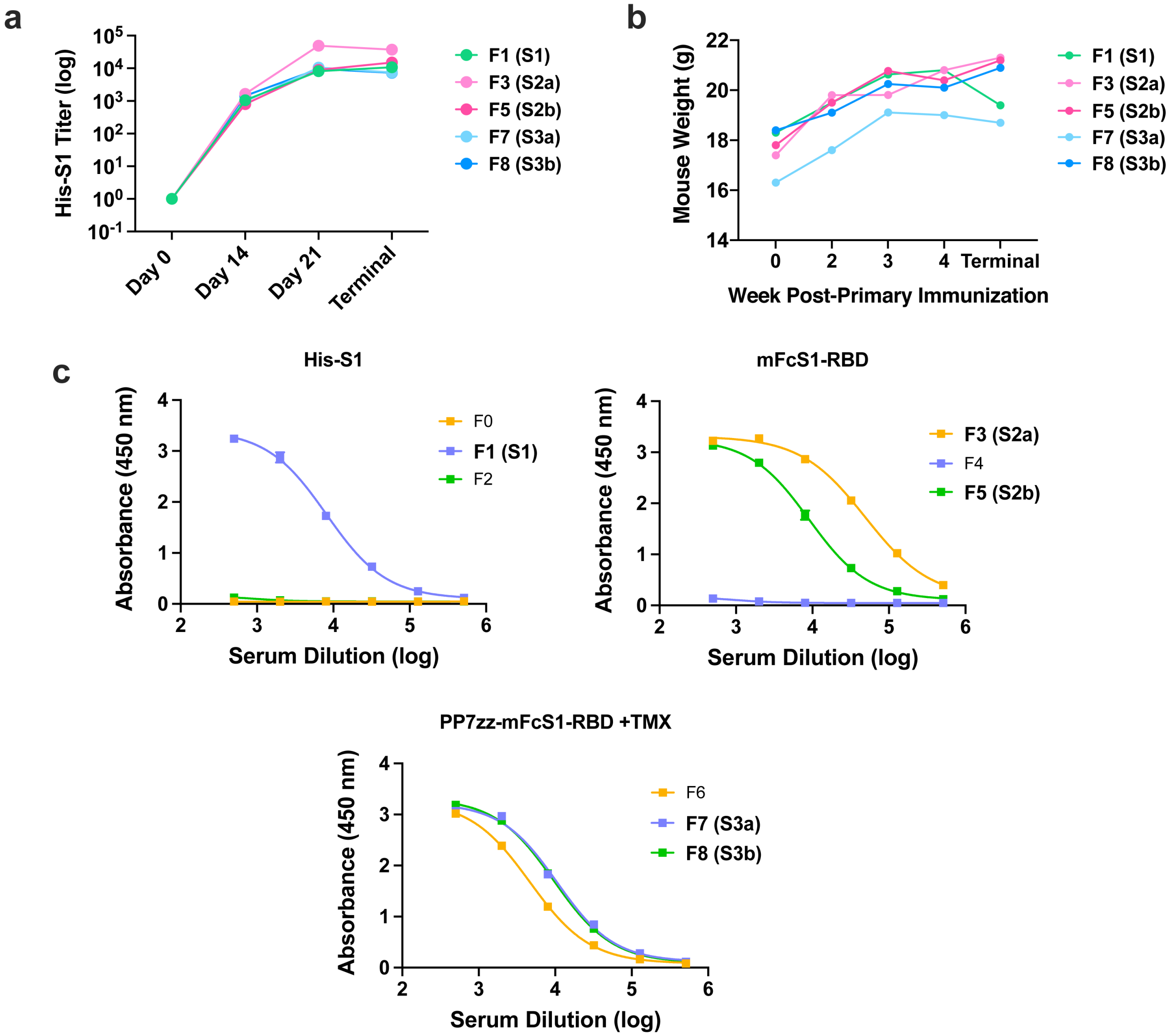

Figure S1. SARS-COV-2 Spike protein subunit vaccine safety and humoral immune response in mice. **a**, Anti-S1 titers over time (fusion mice only) measured by ELISA against His-S1. Day 0 is preimmune sera, Day 14 is two weeks after prime (blood taken prior to boost 1), Day 21 is one week after the first boost, Terminal sera is Day 30. **b**, Fusion mouse weight over the course of vaccine schedule (n=5). **c**, Vaccine response at day 21 as assessed by ELISA. Immunized mouse sera from each group was collected, diluted, and tested against His-S1 (1 µg/mL) (F1: His-S1 + Titermax Gold [Series 1]; F3 and F5: mFcS1-RBD + Titermax [Series 2]; F7-F8: PP7zzmFcS1-RBD + Titermax Gold adjuvant [Series 3]).

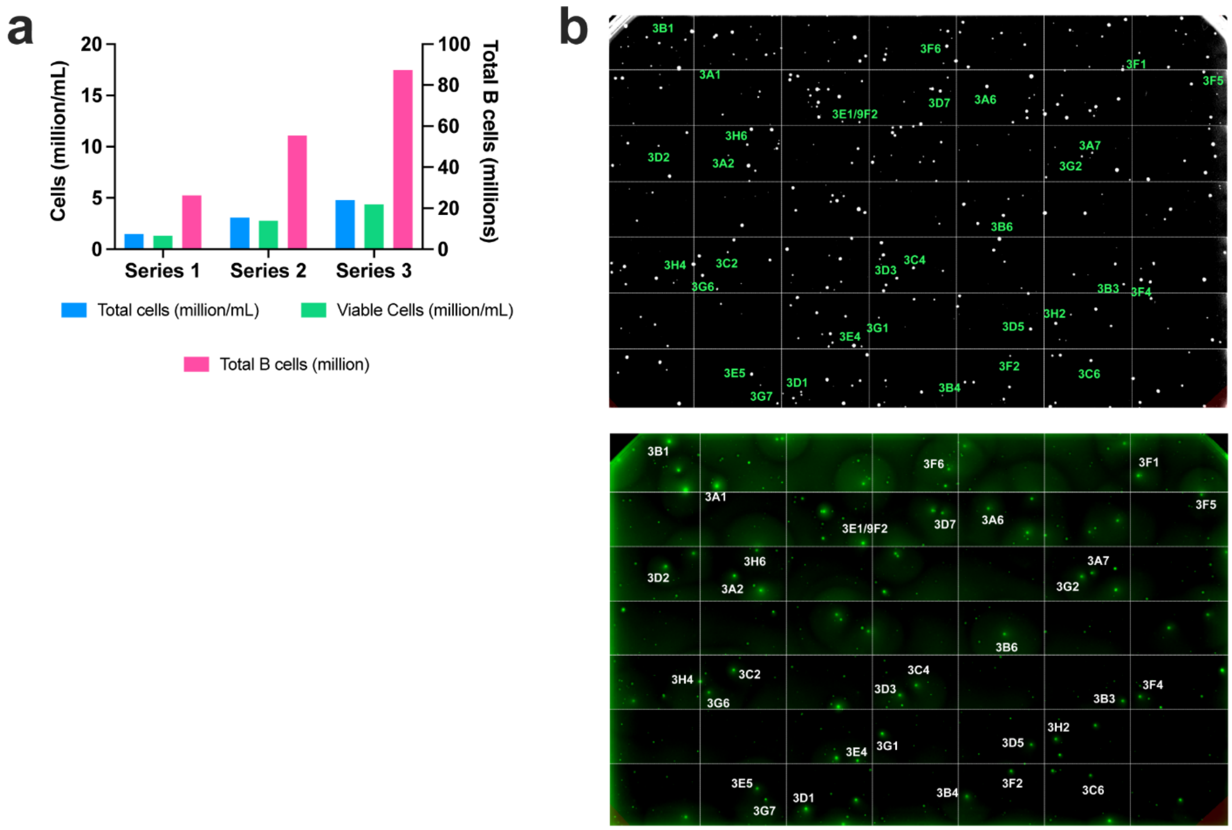

Figure S2. Hybridoma statistics from SARS-COV-2 immunized mice. a, Total cell yield and B cell yield from mice immunized with either His-tag S1 (Series 1), mouse Fc-tagged S1 Receptor Binding Domain (Series 2), or codelivery of mFcRBD by complexation with PP7zz VLPs (Series 3), all in emulsion with Titermax Gold. **b**, Representative images from 3D culture of IgG secreting hybridoma from the combined B cells harvested from two mice immunized with mFcRBD. (*top*) brightfield, (*bottom*) anti-IgG FITC. Clones were chosen by ClonePix2 based on morphology of each clone and the diameter and brightness of the FITC halo, indicating the level of IgG secretion. IgG secretion meausured using CloneDetect anti-IgG-FITC reagent (Mouse IgH (H+L) specific fluorescein-PN) and detected by ClonePix II instrument.

Table S1. Hybridoma statistics from SARS-COV-2 immunized mice.

[Fusion #], initial immunogen, (mice used)	Total B cells fused	Clones detected (fusion efficiency)	High/Low FITC clones detected (%)	Clones spike reactive (total clones picked)	Clones dual antigen reactive	Clones with ecto preference	Clones with S1 reactive preference
[1] SARS-COV-2 His S1 (mouse F1)	25 x 10^6	699 (2.8×10^{-5})	16/304 (5.2%)	27 (282)	15	3	9
[2] SARS-COV-2 mFcRBD (mice F3 + F5)	55	667 (1.2×10^{-5})	85/184 (46.1%)	157 (258)	107	48	2
[3] SARS-COV-2 PP7zzmFcRBD + TiterMax (mice F7 + F8)	75	307 (0.41×10^{-5})	4/8 (50%)	2 (12)	1	1	0
**Totals**	**155**	**1673 clones**	**105/496 (21.2%)**	**186 (552)**	**123**	**52**	**11**

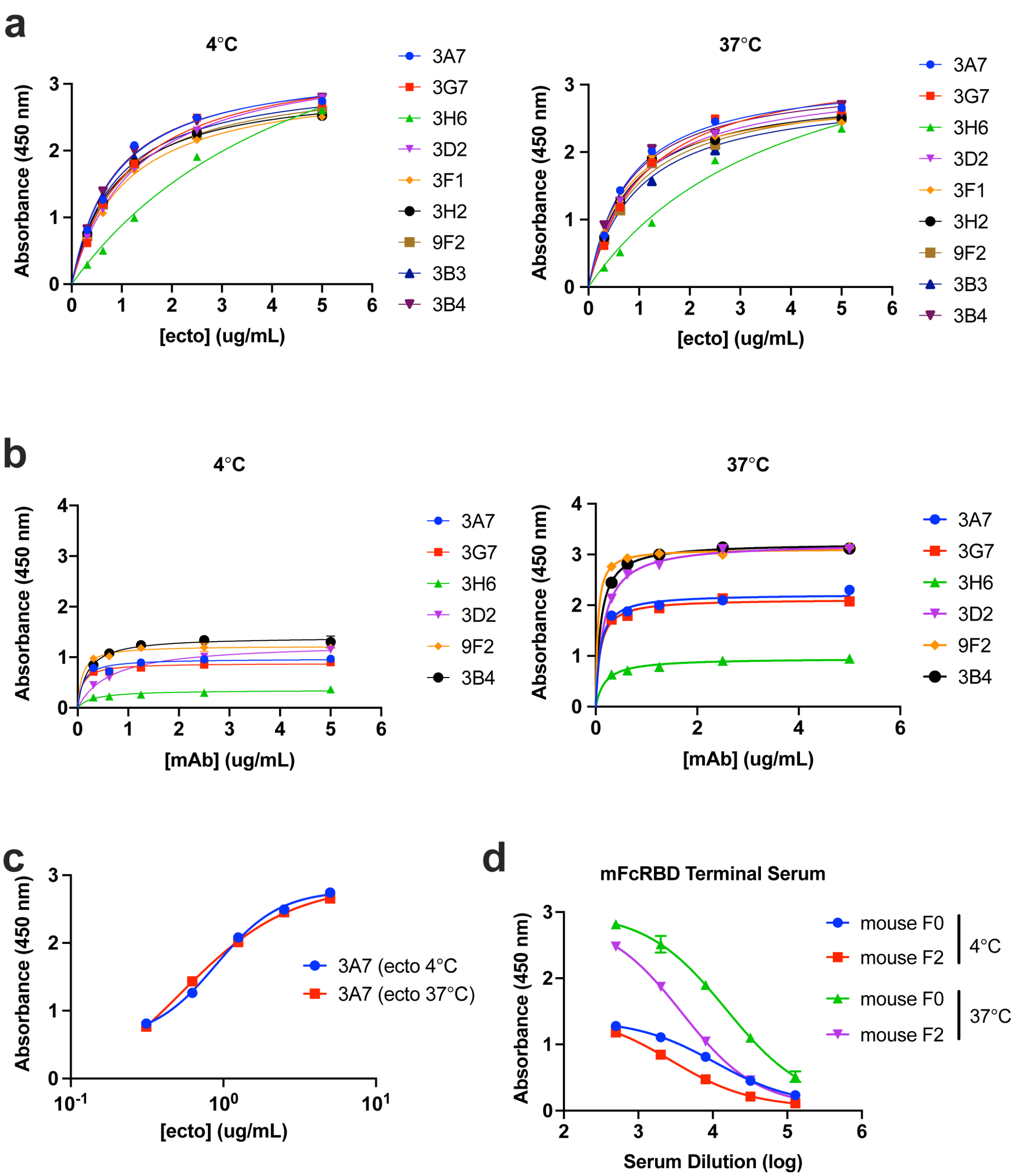

Figure S3. Thermal stability of SARS-CoV-2 spike ectodomain. a, Decreasing concentrations of r-spike ecto protein were plated on streptavidin coated 96-well plates (5 ug/mL), with the protein either used directly from storage at 4°C or after 1 h incubation at 37°C. The indicated monoclonal antibodies were then introduced (10 µg/mL) and incubated for 1 h. After washing, goat anti-mouse IgG-HRP reporter (1:2000 dilution) was added for 1 h, and plates were developed with Ultra-TMB substrate (30 s) and quenched with 2N H_2SO_4. **b**, After equilibration at 4°C or 37°C for one hour, r-spike ecto was plated at 0.5 µg/mL for one hour followed by incubation with decreasing concentrations of select mAbs (5 µg/mL – 0 µg/mL) under the conditions described for panel (**a**). **c**, Comparative binding profile of anti-RBD mAb 3A7 to r-spike ecto plated after temperature equilibration at either 4°C or 37°C. **d**, Terminal sera ELISA from mice immunized with mFcRBD (from which 32 out of 33 selected mAbs originated) against temperature-varied r-spike ecto (0.5 µg/mL).

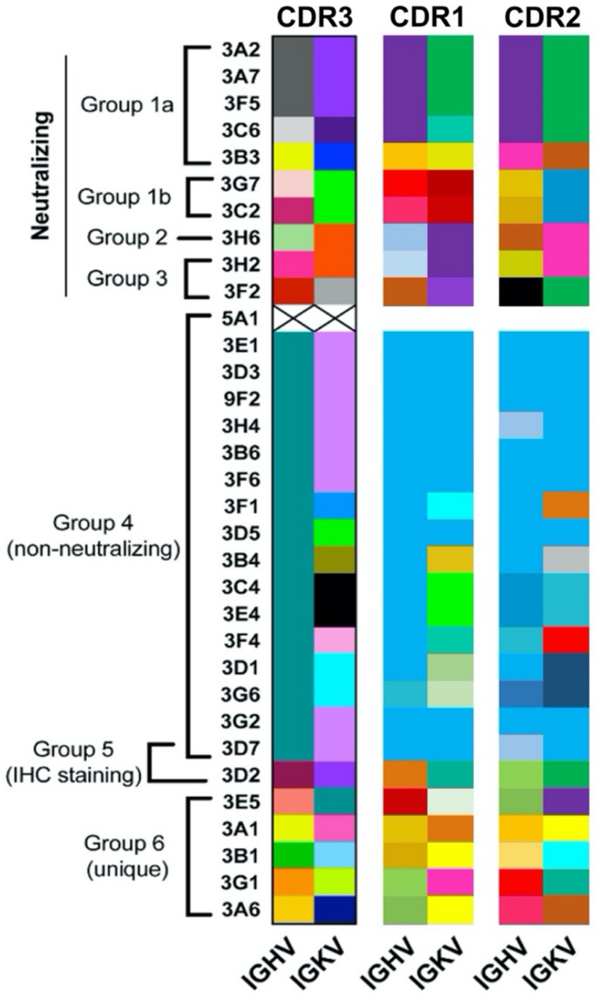

Figure S4. Sequence relationships in complementarity-determining regions. CDR3 portion is shown in Figure 3e of the main paper and represents gene usage. Color coding for CDR1 and CDR2 is based on sequence similarity for these much shorter regions (generally, 3-12 amino acids). Closely related sequences for each CDR are indicated by colors with similar hue. No similarity is implied by identical or related colors between columns. Unique mAbs are unique across CDRs, mAbs clustered by CDR3 maintain those trends across CDRs as well. See Tables S2 and S3 for CDR sequences.

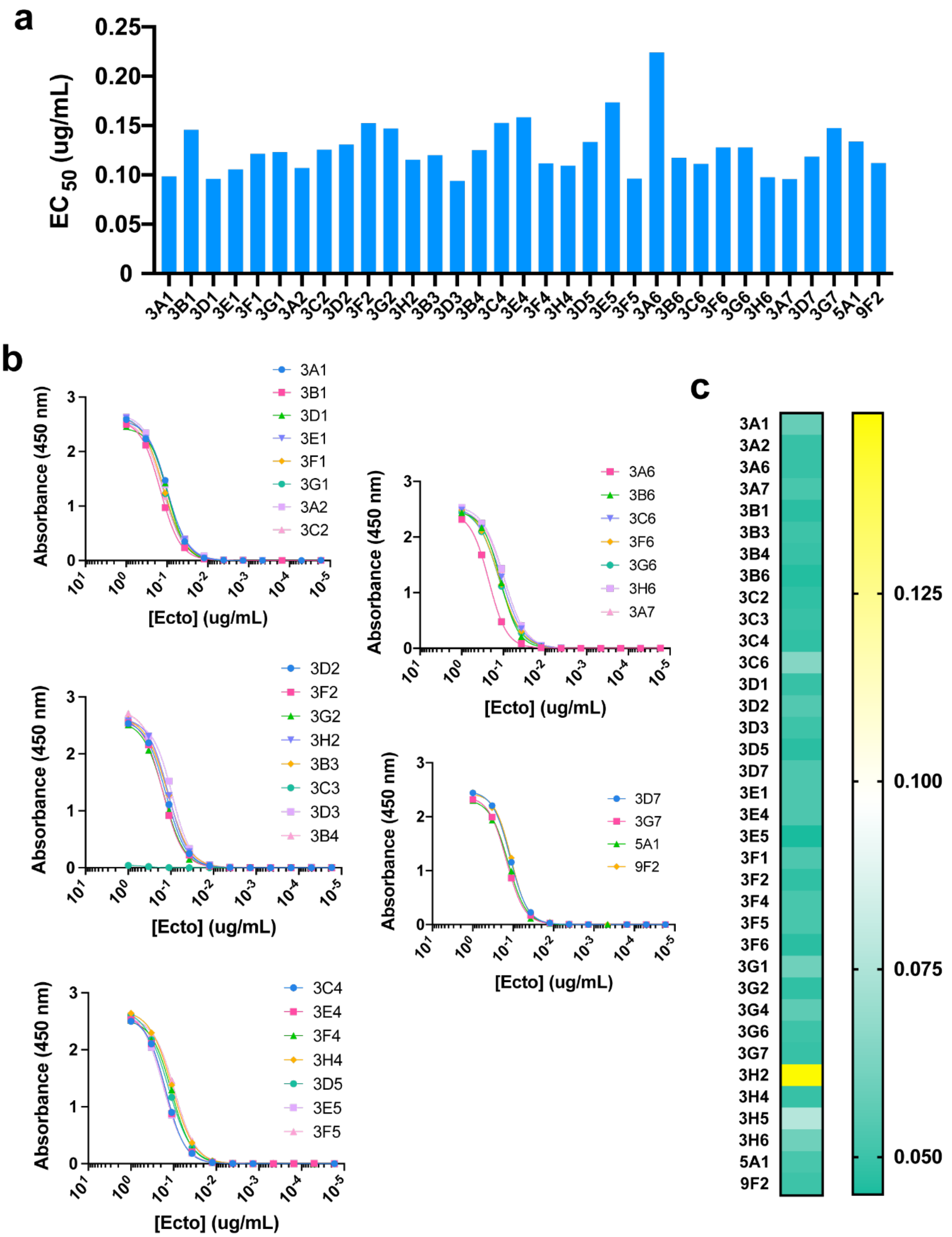

Figure S5. EC50 values for binding to plated r-spike ecto. a, EC50 (µg/mL) of SARS-CoV-2 mAbs to trimeric spike ectodomain, calculated using EC50 shift nonlinear regression (GraphPad Prism v.8) from plots in panel (**b**). **b**, Indirect ELISA mAb (1 µg/mL) sensitivity to spike ectodomain (titration beginning at 1 µg/mL). **c**, Qualitative response (optical density) from indirect ELISA of mAbs against plated heat-inactivated SARS-CoV-2 (obtained from ATCC).

Table S2. Sequences and amino acid lengths of heavy chain CDRs for 32 SARS-CoV-2 monoclonal antibodies. mAbs were sequenced by Illumina next generation sequencing, and heavy and light chain variable gene and CDR3 regions assigned by MiXIR software. Corresponding epitope bins are included, as determined by competitive BLI and summarized in Figure S5.

		Heavy Chain Sequence					
**mAb**	**Bin**	**CDR1**	length	**CDR2**	length	**CDR3**	length
3A2	***1a***	GFSLSTSGMG	10	IYWDDDK	7	CARRGPLITTDGTFDVW	17
3A7	***1a***	GFSLSTSGMG	10	IYWDDDK	7	CARRGPLITTDGTFDVW	17
3F5	***1a***	GFSLSTSGMG	10	IYWDDDK	7	CARRGPLITTDGTFDVW	17
**3C6**	**1a**	GFSLSTSGMG	10	IYWDDDK	7	CARRGPLITTDGTFDVW	17
**3B3**	**1a**	GYSITSDYA	9	ISYSGTT	7	CAGRRGAYYGNYEEDYW	17
**3G7**	**1b**	GYSFTSYW	8	IHPSDSET	8	CARSSGYDWYFDVW	14
**3C2**	**1b**	GYAFTSYW	8	IYPGDGAT	8	CARSGGYDWYFDVW	14
**3H6**	**2**	GFTFNTYA	8	IRSKSDNFAM	10	CASYDGYRAWFAYW	14
**3H2**	**3**	GNTFSNYW	8	ILPGSDST	8	CARNRFYWYFDVW	13
**3F2**	**3**	GYTFTSYS	8	VYPGNDDT	8	CARDGYFAMDYW	12
3E1	***4***	GFNIKDTY	8	IDPANGNT	8	CARTYYYGSSYEAMDYW	17
3D3	***4***	GFNIKDTY	8	IDPANGNT	8	CARTYYYGSSYEAMDYW	17
9F2	***4***	GFNIKDTY	8	IDPANGNT	8	CARTYYYGSSYEAMDYW	17
**3H4**	**4**	GFNIKDTY	8	IDPANGNS	8	CARSYYDYDGGGCFDYW	17
**3B6**	**4**	GFNIKDTY	8	IDPASGNT	8	CARSYYTYDGFFDVW	15
**3F6**	**4**	GFNIKDTY	8	IDPASGNT	8	CARSYYTYDGFFDVW	15
**3F1**	**4**	GFNIKDTY	8	IDPANGNT	8	CVSGYYYYGSPYGAMDYW	18
**3D5**	**4**	GFNIKDTY	8	IDPANGNT	8	CTRYYYGSSGFFDVW	15
**3B4**	**4**	GFNIKDTY	8	IDPANGDT	8	CARSYYYGTTSWFASW	16
3C4	***4***	GFNIKDTY	8	IDPASGKT	8	CASGYDVNYELDYW	14
3E4	***4***	GFNIKDTY	8	IDPASGKT	8	CASGYDVNYELDYW	14
**3F4**	**4**	GFNIKDTY	8	IDPANDNT	8	CTRYYDYVYAMDYW	14
**3D1**	**4**	GFNIKDTY	8	IDPANGNT	8	CARWDFGNYVDYAMDYW	17
**3G6**	**4**	GFNIKDTN	8	IDPANGDT	8	CARLNYDGYYDYAMDYW	17
**3G2**	**4**	GFNIKDTY	8	IDPANGNT	8	CTRYYYGSSGFFDVW	15
**3D7**	**5**	GFNIKDTY	8	IDPANGNS	8	CARSYYDYDGGGCFDYW	17
**3D2**	**5**	GYTFSTYW	8	INPYTDYT	8	CARRYGNYDAWFTYW	15
**3E5**	**6**	GYSFTGYF	8	INPYNGDT	8	CGLRTYW	7
**3A1**	**6**	GYSITGDYS	9	IHYSGSA	7	CARWGNGKNAMDYW	14
**3B1**	**6**	GYSITSGYY	9	IIYDGTN	7	CARVDYDVGHWFAYW	15
**3G1**	**6**	GFSLTSYG	8	IWSGGST	7	CAKYRYDSFAYW	12
**3A6**	**6**	GFSLISYG	8	IWAGGST	7	CGRDYGILLIDYW	13

Table S3. Sequences and amino acid lengths of light chain CDRs for 32 SARS-CoV-2 monoclonal antibodies. mAbs were sequenced by Illumina next generation sequencing, and heavy and light chain variable gene and CDR3 regions assigned by MiXIR software. Corresponding epitope bins are included, as determined by competitive BLI and summarized in Figure S5.

		Light Chain Sequence					
**mAb**	**Bin**	**CDR1**	length	**CDR2**	length	**CDR3**	length
3A2	***1a***	QDVGTS	6	WAS	3	CQQYSSYPYTF	11
3A7	***1a***	QDVGTS	6	WAS	3	CQQYSSYPYTF	11
3F5	***1a***	QDVGTS	6	WAS	3	CQQYSSYPYTF	11
**3C6**	**1a**	QDVDTA	6	WAS	3	CQQYSSYPYTF	11
**3B3**	**1a**	SIVNY	5	DTS	3	CQQWSSYPYTF	11
**3G7**	**1b**	ESVDSYGNSF	10	RAS	3	CQQSNEDPWTF	11
**3C2**	**1b**	ETVDSYGNSF	10	RAS	3	CQQSNEDPWTF	11
**3H6**	**2**	QSLVHSNGNTY	11	KVS	3	CSQSTHVPWTF	11
**3H2**	**3**	QSLVHSNGNTY	11	KVS	3	CSQSTHVPWTF	11
**3F2**	**3**	QSLLYSTNQKNY	12	WAS	3	CHQYYSYPWTF	11
3E1	***4***	QSVSND	6	YAS	3	CQQDYSSPTF	10
3D3	***4***	QSVSND	6	YAS	3	CQQDYSSPTF	10
9F2	***4***	QSVSND	6	YAS	3	CQQDYSSPTF	10
**3H4**	**4**	QSVSND	6	YAS	3	CQQGYSSPLTF	11
**3B6**	**4**	QSVSND	6	YAS	3	CQQDYSSPPTF	11
**3F6**	**4**	QSVSND	6	YAS	3	CQQDYSSPPTF	11
**3F1**	**4**	SSVSY	5	HTS	3	CQQYHGYPLTF	11
**3D5**	**4**	QSVSND	6	YAS	3	CQQDYSSPTF	10
**3B4**	**4**	EDIYNH	6	GAP	3	CQQYWSTPYTF	11
3C4	***4***	QNINVW	6	KAS	3	CQQGQSYPYTF	11
3E4	***4***	QNINVW	6	KAS	3	CQQGQSYPYTF	11
**3F4**	**4**	QTIGTW	6	AAT	3	CQQLYSTPLTF	11
**3D1**	**4**	QDLYSF	6	RAN	3	CLQYDAFPWTF	11
**3G6**	**4**	QDINSF	6	RAN	3	CLQYDEFPFTF	11
**3G2**	**4**	QSVSND	6	YAS	3	CQQDYSSPTF	10
**3D7**	**5**	QSVSND	6	YAS	3	CQQGYSSPLTF	11
**3D2**	**5**	QDVGTA	6	WAS	3	CQQYRTF	7
**3E5**	**6**	QDINNN	6	HGT	3	CVQSVQFPYTF	11
**3A1**	**6**	ENIYSY	6	NTK	3	CQHHYGSPPTF	11
**3B1**	**6**	SSVNY	5	YTS	3	CHQFTTSPWTF	11
**3G1**	**6**	KSVSTSGYSY	10	LAS	3	CQHSRELPYTF	11
**3A6**	**6**	SSVSY	5	DTS	3	CFQGSGYPFTF	11

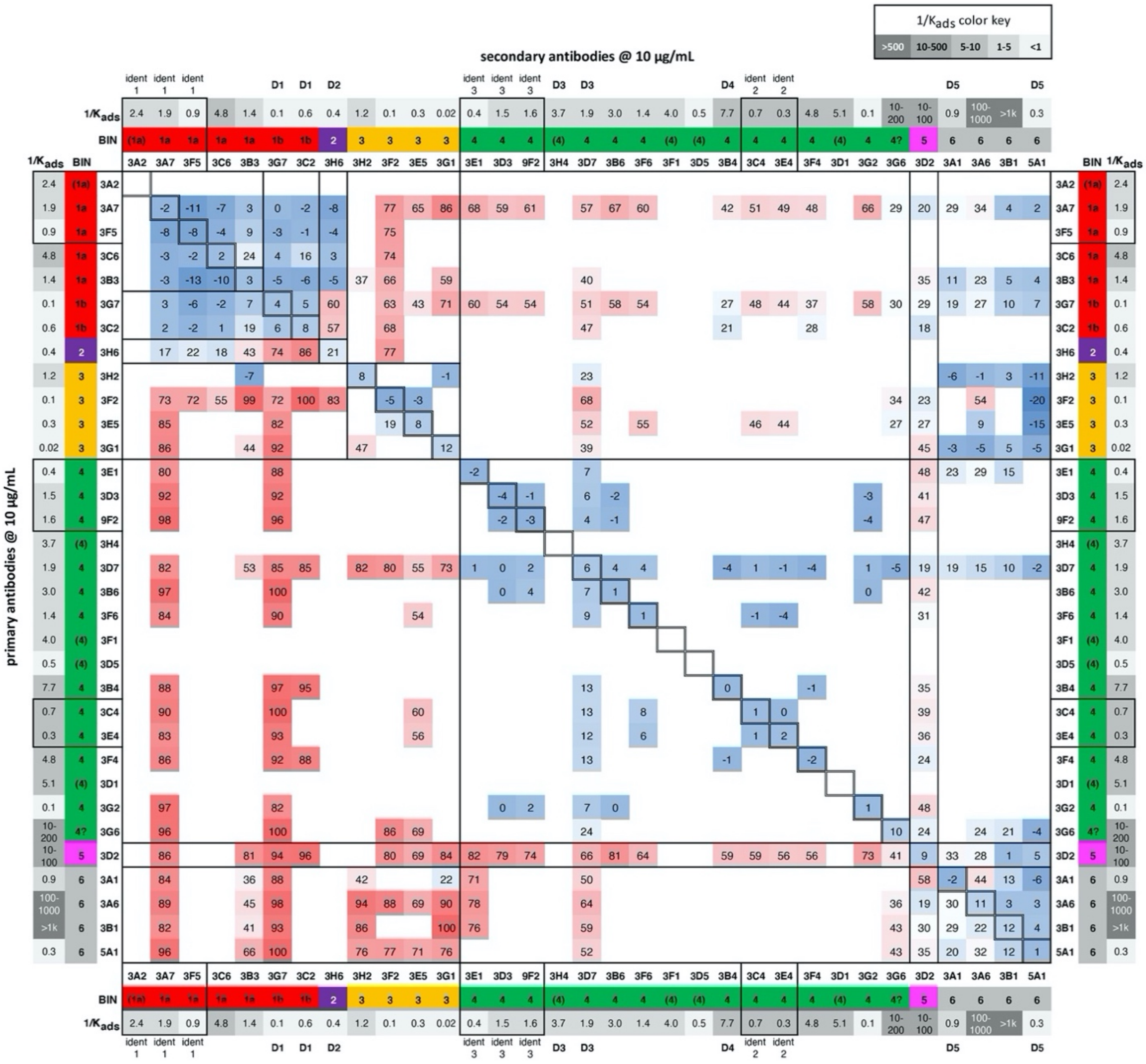

notable disconnections between binding and split-luciferase results

class	mAbs	label	binding to full-length ACE2	binding to truncated ACE2	split luc SARS-1	split luc SARS-2
1b	3G7,3C2	D1	mod. Inhib	enhanced	little effect	little effect
2	3H6	D2	inhib	inhib	little effect	little effect
4	3H4,3D7	D3	little effect	enhanced	little effect	little effect
4	3B4	D4	mod. Inhib	enhanced	enhanced	enhanced
6	3A1,5A1	D5	little effect	little effect	little effect	mod-strong inhib

Figure S6. Collected from competitive binding experiments of the type shown in Figure 3f and Figure S6, performed on groups of up to 13 mAbs at a time. BLI values for each experiment were normalized to a maximum of 100 to allow for incorporation into this master dataset. Color-coded bin assignments are the same as done by sequence. When rows do not match columns, the avidities of the antibodies were usually significantly different, skewing the results of this competitive binding assay. Blank squares denote comparisons not made. Most of group 6 (5A1, 3B1, 3G4, 3C3, 3H5, 9B1) are included because some data was collected, but they were not sequenced and are omitted from the list of 33 selected antibodies discussed in the main text. The three sets of identical antibodies are denoted by "ident #". The table at the bottom summarizes results described in the text concerning the experiments shown in Figure 4a vs. 4f, highlighting the connection between competitive binding, functional properties, and sequence.

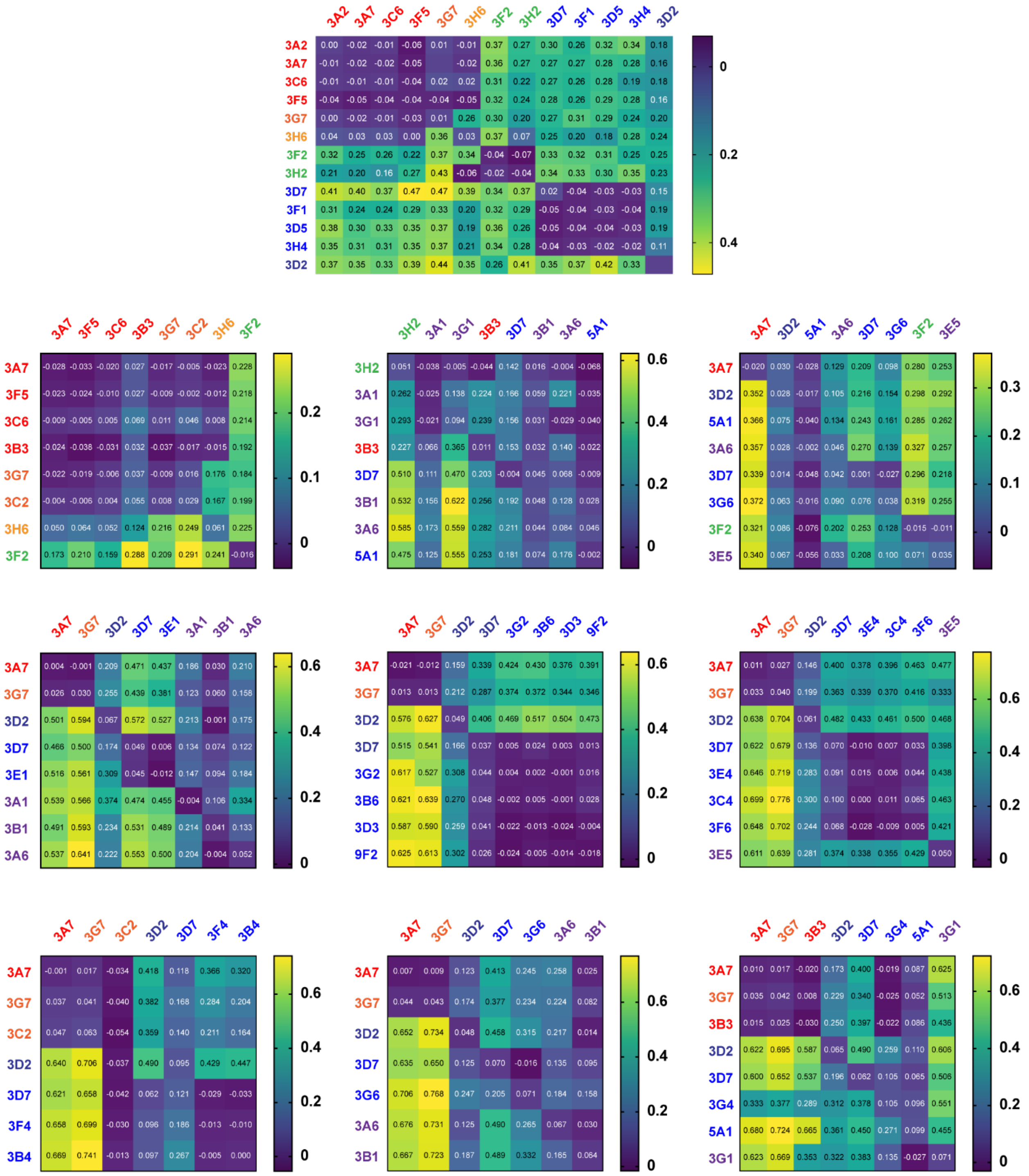

Figure S7. Individual epitope binning results. Epitope classification experiments performed by BLI. r-Spike ectodomain protein (bearing twin Strep-Tag II sequences) was immobilized on streptavidin-coated biosensors (10 µg/mL). Primary antibody (y axis) was incubated for 400 s, allowed to dissociate in buffer for 300 s, followed by incubation with secondary antibody (x axis) for 300 s. Data analyzed using ForteBio Octet Data Analysis software v. 10.0.
